# Juvenile Hormone Studies in *Drosophila melanogaster*

**DOI:** 10.3389/fphys.2021.785320

**Published:** 2022-02-10

**Authors:** Xiaoshuai Zhang, Sheng Li, Suning Liu

**Affiliations:** ^1^Guangdong Provincial Key Laboratory of Insect Developmental Biology and Applied Technology, Institute of Insect Science and Technology, School of Life Sciences, South China Normal University, Guangzhou, China; ^2^Guangdong Provincial Key Laboratory of Insect Developmental Biology and Applied Technology, Guangmeiyuan R&D Center, South China Normal University, Meizhou, China

**Keywords:** juvenile hormone, corpus allatum, methoprene-tolerant, *Drosophila melanogaster*, metamorphosis, reproduction, behavior

## Abstract

In the field of insect endocrinology, juvenile hormone (JH) is one of the most wondrous entomological terms. As a unique sesquiterpenoid hormone produced and released by the endocrine gland, corpus allatum (CA), JH is a critical regulator in multiple developmental and physiological processes, such as metamorphosis, reproduction, and behavior. Benefited from the precise genetic interventions and simplicity, the fruit fly, *Drosophila melanogaster*, is an indispensable model in JH studies. This review is aimed to present the regulatory factors on JH biosynthesis and an overview of the regulatory roles of JH in *Drosophila.* The future directions of JH studies are also discussed, and a few hot spots are highlighted.

## Introduction

Juvenile hormone (JH) primarily produced and secreted from the corpus allatum (CA), fulfills essential roles in many aspects of insect physiology. JH was originally discovered by Wigglesworth (1934) in the triatomine bug, *Rhodnius prolixus*, to be associated with regulating metamorphosis. Subsequently, JH has been extensively studied in insects, where multiple physiological processes are demonstrated to be controlled by JH, such as reproduction, caste determination and differentiation, diapause, immunity, aging, and behavior (Goodman and Cusson, 2012; Rivera-Perez et al., 2020).

Unlike some holometabolous insects, the application of JH or JH analogs to the fruit fly, *Drosophila melanogaster* (*D. melanogaster*), exhibits prolongation of the final larval instar or lethality during pupal-adult transition instead of causing extra larval molting (Bryant and Sang, 1968; Ashburner, 1970; Madhavan, 1973; Postlethwait, 1974; Riddiford and Ashburner, 1991). Despite the disadvantage of studying JH on metamorphosis, *Drosophila* has been developed as a powerful model system to investigate molecular mechanisms of JH action on a diverse range of biological processes, by capitalizing on a vast array of powerful genetic and molecular approaches (Noriega, 2014; Li K. et al., 2019; Riddiford, 2020). This review attempts to provide an overview of JH research in *Drosophila* and outline the potential of this organism to understand hormonal regulation of insect development.

## Juvenile Hormone Metabolism

### Corpus Allatum

The *Drosophila* CA, which originates from the migration of ectodermal cells in the maxilla and labium during embryogenesis controlled by Hox proteins (Sánchez-Higueras et al., 2014), is an anteromedial subtissue of the ring gland adjacent to the prothoracic gland (PG) and corpora cardiac (CC) during the larval stage (King et al., 1966). CA cells are maintained to be the progenitor of the adult CA, whereas PG is broken down during metamorphosis (Dai and Gilbert, 1991). The mitochondria and smooth endoplasmic reticulum (SER) in the CA cells are considered to be major organelles involved in JH biosynthesis (King et al., 1966; Dai and Gilbert, 1991). CA cell size is proportional to the number of cellular components, which are likely correlated with the production capacity of JH, but the only increase of CA size might not be the principal factor for JH biosynthesis (Zhang J. et al., 2021).

Studies have reported that JH production by the CA maximizes at the larval stage, then declines dramatically after pupariation, sustains a low level in the inactive CA cells of pupa, and increases again after adult emergence (Richard et al., 1989a,b; Altaratz et al., 1991; Dai and Gilbert, 1991). Over 2 days after eclosion, the JH titer appears to be decaying once again (Zhang S. X. et al., 2021). Even so, it is also necessary to perform more accurately qualitative analysis to study the JH biosynthesis in the CA at the different timeline that relies on the development of new technologies (Rivera-Perez et al., 2012). The *Aug21-Gal4* is a CA-specific driver (Siegmund and Korge, 2001), which was used for either complete (Liu et al., 2009; Riddiford et al., 2010) or incomplete genetic ablation (Gruntenko et al., 2010, 2012; Bilen et al., 2013; Yamamoto et al., 2013), that impaired JH biosynthesis. The destruction of the CA for manipulating endogenous JH allowed us to examine the role of JH during different stages.

### Juvenile Hormone Biosynthesis

In *Drosophila*, three sesquiterpenoid products, methyl farnesoate (MF), JH III, and the principal form JH bisepoxide (JHB3), are produced in the CA via the mevalonate pathway (Richard et al., 1989a,b; Bellés et al., 2005; Harshman et al., 2010; Jones et al., 2010; Wen et al., 2015). To date, though the entire JH biosynthetic pathway in *Drosophila* has not been fully defined, the majority of enzymes have been characterized in the steps from acetyl-CoA to JHs.

Juvenile hormone biosynthesis involves multiple enzymatic catalytic reactions and is conventionally divided into early and late steps (Bellés et al., 2005). The early steps follow the mevalonate pathway to form farnesyl pyrophosphate (FPP). The 3-hydroxy-3-methylglutaryl-CoA synthase (HMG-S), 3-hydroxy-3-methylglutaryl CoA reductase (HMGCR), and Farnesyl diphosphate synthase (FPPS) are important regulatory enzymes in the steps of FPP formation (Bellés et al., 2005). In the late steps, farnesoic acid (FA) is converted to MF, JH III, and JHB3. JH acid methyltransferase (JHAMT) is identified as a rate-limiting enzyme that converts FA and JH acid to MF and JH III in insects via *in vitro* assays (Shinoda and Itoyama, 2003; Niwa et al., 2008; Defelipe et al., 2011). Interestingly, knockout or overexpression of *Jhamt* in the *Drosophila* CA has no effect on MF or JH III biosynthesis but alters JHB3 titer *in vivo* (Bendena et al., 2011; Wen et al., 2015), suggesting that JHAMT is only responsible for JHB3 biosynthesis in *Drosophila*. Alternately, JHB3 is synthesized by a P450-mediated epoxidation reaction (Moshitzky and Applebaum, 1995). Cytochrome P450 6g2 (Cyp6g2) has emerged as a promising candidate owing to the performance of *in situ* hybridization and RNAi experiments (Chung et al., 2009; Christesen et al., 2017), while a *Cyp6g2* null allele and a *Jhamt Cyp6g2* double mutant are urgently required for the understanding JH biosynthesis in *Drosophila*.

### Regulation of Juvenile Hormone Biosynthesis

Nowadays, compared to the classical insect model in JH earlier research, *Drosophila* with powerful genetic manipulation has become the leader in the search for the regulatory mechanism of JH biosynthesis (Noriega, 2014). In the past 20 years, much progress has been made in understanding the regulation of JH biosynthesis, and various factors have been identified in *Drosophila*, such as insulin (Tatar et al., 2001; Tu et al., 2005; Belgacem and Martin, 2007), ecdysteroids (Liu et al., 2018), miRNAs (Qu et al., 2017; Zhang J. et al., 2021), biogenic amines (Chiang et al., 2002; Gruntenko et al., 2005, 2007; Huang et al., 2011), Decapentaplegic (Dpp) (Huang et al., 2011), Ecdysis-triggering hormone (ETH) (Meiselman et al., 2017, 2018), and sex peptide (SP) (Moshitzky et al., 1996; Bontonou et al., 2015; Schwenke and Lazzaro, 2017). In essence, JH biosynthesis by the CA is considered to be controlled at the level of the expression of JH biosynthetic enzymes (Hiruma and Kaneko, 2013). Nevertheless, the transcriptional regulatory mechanism of JH biosynthesis is nearly unknown in other insects, *Drosophila* might be a useful tool to make breakthroughs in this direction.

In insects, nutrition, via the insulin/insulin-like growth factor (IIS)/target of rapamycin (TOR) signaling pathway, mediates its effect on body size partially by regulating JH biosynthesis at specific points during development (Koyama et al., 2013; Zhu et al., 2020). Mutation of *Drosophila insulin receptor* (*InR*) decreases JH biosynthesis (Tatar et al., 2001; Tu et al., 2005). Likewise, CA-specific silencing of *InR* suppresses *Hmgcr* expression (Belgacem and Martin, 2007), while ectopic activation of the PI3K is sufficient to promote *Jhamt* expression and CA cell growth (Zhang J. et al., 2021). In response to starvation, increased levels of ecdysteroids, especially 20-hydroxyecdysone (20E), have been demonstrated to negatively regulate JH biosynthesis (Terashima et al., 2005; Meiselman et al., 2017, 2018). Moreover, inhibition of 20E signaling in the CA leads to elevated JH biosynthesis by upregulating *Jhamt* and *Hmgcr*, which, in turn, prevents ecdysone biosynthesis in the PG and 20E-induced metamorphosis (Liu et al., 2018; Zhang et al., 2018).

MicroRNAs are a major group of small endogenous non-coding RNAs that act as post-transcriptional regulators of JH biosynthesis and subsequent JH signaling (Qu et al., 2017, 2018). By using high-throughput sequencing, the expression profiles of *Drosophila* microRNAs have been identified in the ring gland. In combination with the performance of a two-tiered screening approach, miR-8 has been identified as a positive regulator of CA growth and JH biosynthesis (Zhang J. et al., 2021). In addition, over-expression of Bantam using the CA-specific drivers results in the inhibition of *Jhamt* expression, a decrease of JH titer, and pupal lethality (Qu et al., 2017; Zhang J. et al., 2021). The research studies on JH biosynthesis regulated by microRNAs are still in the initial stage and worth exploring in the future.

Neurons can directly innervate the CA to affect JH biosynthesis by releasing neurotransmitters, in particular, biogenic amines (Siegmund and Korge, 2001; Chiang et al., 2002; Bendena et al., 2020). For example, dopamine influences JH production (Gruntenko et al., 2005, 2007). Moreover, glutamate binds to and activates *N*-methyl-D-aspartate (NMDA) receptors in the *Drosophila* CA (Chiang et al., 2002), and activation of the NMDA signaling in the CA indirectly stimulates JH biosynthesis through Dpp signaling-mediated *Jhamt* expression (Huang et al., 2011). The regulation of JH production also occurs through the actions of neuropeptides (Bendena et al., 2020). Allatostatin-C (AST-C) acts on its receptor (AST-CR1 and AST-CR2) in the CA to inhibit JH biosynthesis (Wang et al., 2012). Alternatively, the neuropeptide ETH, released by endocrine Inka cells, stimulates JH biosynthesis through action on CA in which the ETH receptor gene is expressed (Meiselman et al., 2017). At the adult stage, the male SP with sperm is transferred to the female during mating, and then SP activates JHB3 production in the CA (Moshitzky et al., 1996; Bontonou et al., 2015; Schwenke and Lazzaro, 2017). However, it should be noted that a subset of neurons directly projected to the adult CA might not participate in the regulation of JH biosynthesis, such as hugin neurons (Mizuno et al., 2021).

Over the years, we have learned nothing about how the expression of JH biosynthetic enzymes is regulated by transcription factor (TF) in *Drosophila* except for apterous (ap) (Postlethwait and Weiser, 1973; Postlethwait and Jones, 1978; Tompkins, 1990; Altaratz et al., 1991; Ringo et al., 1992; Shtorch et al., 1995). Mutation of *ap* leads to a decrease in JH titer, delayed maturation of adult fat body, and male courtship defects (Tompkins, 1990; Ringo et al., 1992; Shtorch et al., 1995). For further research, integrative approaches, such as transcriptomics, proteomics, and large-scale genetic screens, are promising to identify more TFs implicated in direct the regulation of JH biosynthetic enzymes.

### Juvenile Hormone Degradation

Two JH metabolizing enzymes, JH esterase (JHE), largely present in the hemolymph, and JH epoxide hydrolase (JHEH), found in tissues, have been identified in *Drosophila* (Campbell et al., 1992; Kethidi et al., 2005; Crone et al., 2007). JHE or JHEH causes hydrolysis of the methyl ester or epoxide moiety of JH resulting in the conversion of JH into JH acid or JH diol, respectively (Kamita and Hammock, 2010). The developmental expression levels are quantified and found that JHE mRNA levels increase during JH III peaks in the hemolymph and decrease during ecdysteroid peaks in the hemolymph (Kethidi et al., 2005), suggesting JHE is also controlled by these hormones. Over-expression of a JHE-binding protein resulted in adult phenotypes is associated with decreased JH (Liu et al., 2008). Thus, the balance between JH biosynthesis and degradation is contributed to the stringent regulation of JH, which is essential for normal insect development and metamorphosis.

## Juvenile Hormone Signaling Transduction

### Intracellular Receptor

The discovery of gene *Methoprene-tolerant* (*Met*) by Wilson and Fabian nearly 35 years ago in *Drosophila* (Wilson and Fabian, 1986) was a milestone event for understanding JH signaling transduction, although the key features of *Met* as the JH receptor were underscored in a non-fruit fly model (Konopova and Jindra, 2007). In 1986, the mutation at this locus was obtained by Wilson lab using mutagenesis screen, and they showed that *Drosophila* with loss of *Met* is highly resistant to the toxic effects of JH analog methoprene (Wilson and Fabian, 1986). Unlike the lethality by CA ablation (Liu et al., 2009; Riddiford et al., 2010), *Met* null mutation was viable with subtle defects in phenotypes (Wilson and Fabian, 1986; Ashok et al., 1998; Wilson and Ashok, 1998). It seemed that *Met* encodes a non-vital protein and another gene appears to function redundantly in JH reception (Wilson and Ashok, 1998).

As the members of the basic helix-loop-helix (bHLH)-Per-Arnt-Sim (PAS) family of TFs, *Me*t is derived from the ancestral gene *germ cell-expressed* (*Gce*) (Baumann et al., 2010). Both *Met* and *Gce* bind to JH III and MF and JH analogs with high affinity in the PAS-B domain (Shemshedini and Wilson, 1990; Ashok et al., 1998; Miura et al., 2005; Charles et al., 2011; Jindra et al., 2015b; Bittova et al., 2019). They form homodimers or heterodimers and JH reduces this dimerization (Godlewski et al., 2006). Defective phenotypes, such as precocious and enhanced programmed cell death (PCD) and pupal lethality in *Met/Gce* double mutant, are similar to those found in JH-deficient flies (Liu et al., 2009; Abdou et al., 2011). Importantly, they could be rescued by exogenous JH analog pyriproxyfen in JH-deficient flies but not in *Met/Gce* double mutant (Abdou et al., 2011). The requirement of direct JH-binding capacity to *Met*/*Gce in vivo* for JH action is required for the fly normal development (Jindra et al., 2015b). All findings together demonstrate that *Met* and *Gce* mediate the effects of JH as the intracellular JH receptor.

### Signaling Transduction

Identification of JH receptor accelerates the research studies on JH intracellular signaling transduction in *Drosophila*. In detail, Met heterodimerizes with another bHLH-PAS protein Taiman (Tai; steroid response coactivator, SRC or βFtz-F1 Interacting Steroid Receptor Coactivator, FISC) after binding of JH (Li et al., 2011). βFTZ-F1 is also an essential binding protein of Met/Gce for JH signaling (Dubrovsky et al., 2011; Bernardo and Dubrovsky, 2012). As a TF, Met is predominantly localized in the nuclei of cultured cells (Miura et al., 2005; Greb-Markiewicz et al., 2011) and tissues (Pursley et al., 2000). The chaperone heat shock protein 83 (Hsp83) facilitates this Met nuclear import by physically interacting with its PAS-B and bHLH domains (He et al., 2014). Subsequently, Nucleoporin 358 kD (Nup358) promotes the JH-Met-Hsp83 complex to transport into the nucleus dependent on importin β (He et al., 2017). Finally, the Met-cofactors complex binds to the JH response region (JHRR) directly and regulates the expression of JH response genes (He et al., 2014, 2017). The zinc-finger TF Krüppel-homolog 1 (Kr-h1) acts as an early JH-response gene and is recognized as the anti-metamorphosis factor (Minakuchi et al., 2008; Huang et al., 2011; Liu et al., 2018).

## Physiological Actions of Juvenile Hormone Intracellular Signaling

### Metamorphosis

Juvenile hormone was originally discovered for its capacity to prevent metamorphosis (Wigglesworth, 1934; Riddiford, 2020). The prominent metamorphic events in *Drosophila* include the destruction of most larval structures and tissue remodeling. 20E orchestrates these diverse cellular events, and JH prevents 20E-induced metamorphosis via the JH receptor and Kr-h1, both of which are critical for the normal development of insects (Jindra et al., 2013, 2015a). As the main organ of the intermediate metabolism of insects, the fat body plays a central role in the integration of hormonal signals to regulate metamorphosis (Li S. et al., 2019). For example, Kr-h1 transduces the JH intracellular signal to repress 20E responsive genes, namely, *Broad-complex* (*Br-C*) and *ecdysone-inducible proteins E93* (*E93*), which subsequently inhibit 20E-induced precocious program cell death of the larval fat body (Minakuchi et al., 2008; Abdou et al., 2011; Belles and Santos, 2014; Liu X. et al., 2015). Moreover, precocious fat body cell dissociation was observed in both JH-deficient animals and *Met/Gce* double-mutant animals (Liu et al., 2009; Abdou et al., 2011). Kr-h1 represses matrix metalloproteinases (Mmps) expression and thus prevents fat body cell dissociation during the larval-prepupal transition (Jia et al., 2017). Likewise, JH signaling prevents the precocious formation of adult organs, such as the optic lobe. JH removal by CA ablation resulted in precocious optic lobe development during the prepupal period whereas JH application suppressed this visual system defect (Riddiford et al., 2010, 2018). The direct and transient repression of *Kr-h1* by Orthodenticle (Otd) and Ecdysone receptor (EcR) is required for correct photoreceptor maturation, also exhibiting the anti-metamorphosis activity of Kr-h1 in remodeling neurons (Fichelson et al., 2012). With the generation of genetic tools for JH research (He et al., 2014; Baumann et al., 2017), more functions of JH in target tissues during metamorphosis or other processes will be uncovered.

On the other hand, JH can suppress ecdysone synthesis of the PG *in vitro* and *in vivo* (Richard and Gilbert, 1991; Liu et al., 2018; Zhang et al., 2018). Knockdown of *Kr-h1* in the PG results in precocious metamorphosis and pupal lethality, implying the direct regulatory function on ecdysone synthesis (Danielsen et al., 2016; Liu et al., 2018). Indeed, JH directly targets PG to inhibit ecdysone biosynthesis by reducing steroidogenesis autoregulation, PG size, and expression of the steroidogenic enzymes (Liu et al., 2018; Zhang et al., 2018). At the epigenetic level, JH impairs polycomb repressive complex 2 (PRC2)-mediated histone H3 lysine 27 (H3K27) methylation and thereby induces *hairy* expression, and thus inhibits ecdysone biosynthesis by repressing expression of the steroidogenic enzyme to regulate metamorphosis (Yang et al., 2021). The epigenetic regulatory mechanism of JH action will certainly shed light on hormone regulation in animals.

### Reproduction

Juvenile hormone evolves as a gonadotrophic hormone (Dubrovsky et al., 2002; Riddiford, 2012; Santos et al., 2019), which has been implicated in vitellogenesis and yolk protein uptake in *Drosophila* females (Postlethwait and Weiser, 1973; Bownes, 1989; Saunders et al., 1990; Soller et al., 1999; Riddiford, 2012), larval fat body histolysis (Postlethwait and Jones, 1978; Yamamoto et al., 2013), and male accessory gland protein synthesis (Yamamoto et al., 1988; Shemshedini et al., 1990; Wolfner et al., 1997; Wilson et al., 2003).

Previous studies have shown that incomplete ablation of the CA or mutation of *Jhamt* results in the reduction of JH level with an associated reduction in fecundity and ovary size (Gruntenko et al., 2010; Wen et al., 2015). These reproductive deficiencies are caused by decreases of JH-induced Vg production in the fat body and Vg uptake by the oocytes (Luo et al., 2021) or probably due to reduced germline stem cells (Luo et al., 2020). A recent study reports that the single null mutant of JH receptors, *Met*^27^ or *Gce*^2^.^5^*^K^*, also shows decreased fecundity but with abnormal egg shape and ovary size gradually increase. Subsequently, a novel mechanism for JH-regulated *Drosophila* reproduction is uncovered that JH intracellular signaling induces *Laminin* or *Collagen IV* gene expressions in ovarian muscle or fat body cells, respectively, which are contributed to the assembly of ovarian muscle extracellular matrix (ECM) that is indispensable for ovarian muscle contraction, then ovarian muscle contraction externally generates a mechanical force to promote ovulation and maintain egg shape (Luo et al., 2021).

### Behavior

Besides the roles in metamorphosis and reproduction, JH is also known to play roles in the behaviors of *Drosophila*. After eclosion, JH regulates the maturation of female receptivity by promoting the production of sex pheromone (Manning, 1966; Ringo et al., 1991; Bilen et al., 2013). As for mature males, knockdown of *Jhamt* significantly reduced courtship that could be rescued by the application of JH analogs, suggesting the physiological role of JH in male courtship behavior (Wijesekera et al., 2016). Moreover, JH potentiates the sensitivity of a pheromone sensing olfactory receptor OR47b to maximize courtship success (Lin et al., 2016). The activation of Ca2+/calmodulin-dependent protein kinase I (CaMKI) and CREB-binding protein (CBP) enhances the efficacy of JH in male Or47b neurons to modulate pheromone detection and thereby regulate courtship behavior (Sethi et al., 2019). Interestingly, there is a piece of evidence that JH suppresses mating behavior by activating TF cyclic adenosine 3’,5’-monophosphate (cAMP) response element-binding protein 2 (CREB2) in juvenile males (Zhang S. X. et al., 2021), suggesting the complex regulatory function of JH on courtship behavior. Additionally, JH signaling influences the short-term and long-term courtship memory of males by acting on diverse neural circuits (Lee et al., 2017; Lee and Adams, 2021). Furthermore, JH signaling controls sexual dimorphic behaviors, such as locomotion and sleep (Belgacem and Martin, 2007; Wu et al., 2018; Wu B. et al., 2021). Investigating the JH-regulated sexually dimorphic behaviors emerges as a promising direction in JH studies in *Drosophila.*

Conceivably, the known JH intracellular receptors, Met and Gce, mediate the action of JH on different behaviors but their functions are not fully redundant. For females, JH regulates mating and pheromone production primarily via Met (Bilen et al., 2013). For males, on the one hand, Met is necessary for both normal fertility and courtship behavior through modulating Or47b sensitivity (Wilson et al., 2003; Lin et al., 2016). On the other hand, *Met* expression in dopaminergic (DA) neurons and mushroom body (MB) γ lobe neurons is essential for courtship short-term and long-term courtship memory, respectively (Lee et al., 2017; Lee and Adams, 2021). However, Gce is dispensable for long-term courtship memory (Lee and Adams, 2021). In addition, *Met* mutant increases sleep in both males and females, but *Gce* deletion mutant exhibits sexually dimorphic effects on sleep (Wu et al., 2018, Wu B. et al., 2021). There are lots of possible factors for their different functions, for example, the differentiated subcellular and tissue distribution (Baumann et al., 2017; Kolonko et al., 2020) or the disruption of Met-Gce dimerization by JH (Godlewski et al., 2006; Zhang S. X. et al., 2021).

## Juvenile Hormone Membrane Signaling and Action

Juvenile hormone might rapidly exert non-genomic actions through putative plasma membrane receptors in a wide range of insects’ studies (Jindra et al., 2015a). For example, a potential member(s) of the receptor tyrosine kinase (RTK) family might function as the membrane receptor of JH in Diptera insects, namely, *Drosophila* (Liu P. et al., 2015). This JH-RTK pathway activates the phospholipase C (PLC) pathway, leading to the phosphorylation and activation of calcium/CaMKII and protein kinase C (PKC), which subsequently induce phosphorylation of Met and Tai, thus regulating the activity of JH intracellular signaling (Liu P. et al., 2015; Ojani et al., 2016). However, the study on the JH membrane pathway in *Drosophila* is very limited. About three decades ago, Yamamoto et al. (1988) showed that JH regulates protein synthesis in the male accessory glands by activating the PKC pathway, implying the existence and importance of JH membrane signaling (Yamamoto et al., 1988). Until 2021, using genetics and quantitative phosphoproteomics methods, Gao et al. (2021) discovered that JH phosphorylated ultraspiracle protein (USP) at Ser35 through the RTK-PLC-PKC pathway to maximize 20E signal transduction even in the absence of JH intracellular signaling (Gao et al., 2021). It will be beneficial to identify the JH membrane receptors and advance our understanding of the complex JH signaling network.

## Concluding Remarks

**FIGURE 1:**
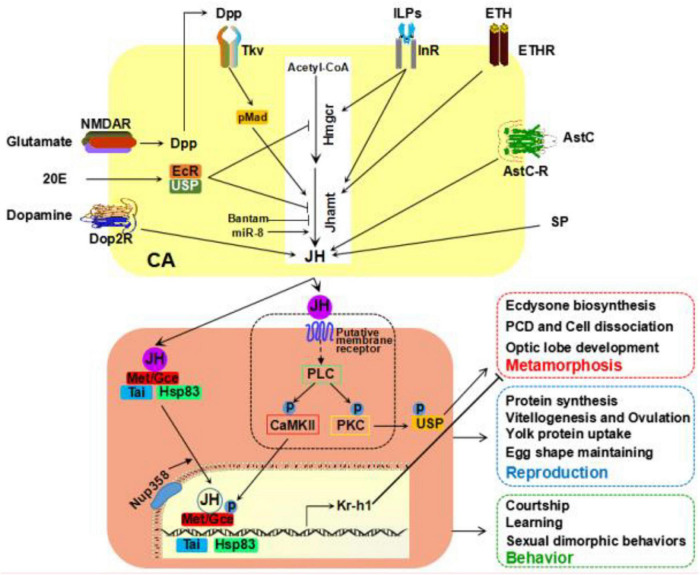
Recent studies on JH studies in *Drosophila*. Examples of various factors affecting JH biosynthesis in corpus allatum (CA) cells at the larval or adult stage. JH employees both intracellular and membrane receptors for signal transduction. JH signaling plays an essential role in multiple physiological processes. JH, juvenile hormone.

Given that the roles of JH are multidirectional and complex, model organisms, such as *Drosophila* and other insects, provide an ideal framework to understand the molecular and cellular mechanisms of JH action regulating insect physiology in response to diverse environmental cues. In this review, we have summarized the knowledge that known factors controlling JH biosynthesis, JH signaling transduction, and its essential impacts on physiological outputs focus on its roles in metamorphosis, reproduction, and behaviors (Figure 1). Based on the accessibility of genetic tools and simplicity of genome, the fruit fly *D. melanogaster* has made great contributions to the field of JH, particularly in the discovery of JH intracellular receptors. Despite that, some questions still need to address in the future.

Although JH action has been investigated mostly in the postembryonic development, its embryonic functions remain unclear. Suppression of JH biosynthesis or JH signaling in *Bombyx mori* or *Tribolium castaneum* results in minor embryonic developmental defects (Shinoda and Itoyama, 2003; Daimon et al., 2015). Conversely, JH signaling is necessary for embryogenesis in some hemimetabolous species, namely, *Blattella germanica* (Fernandez-Nicolas and Belles, 2017). It reveals the complexity of JH action on embryonic development in different species. In *Drosophila*, the larvae can survive up to the end of the larval stage whether they are genetically allatectomized, *Met/Gce* double mutant, or *Jhamt* mutant (Liu et al., 2009; Riddiford et al., 2010; Abdou et al., 2011; Wen et al., 2015). It seems that JH signaling is unimportant for embryonic or even early larval development in this species. However, a recent study reports that JH signaling is activated in mid-embryogenesis prior to CA development, and JH is required for migrating germ cells to reach the somatic gonad via a non-canonical pathway (Barton et al., 2021). Despite JH embryonic functions are relatively minor, *Drosophila* is still a powerful model to explore how JH affects embryonic development.

Our current understanding of JH actions is largely based on phenotypic defects induced by JH treatment or removal, and mutations or RNAi of JH signaling, which have limitations to analyze the JH functions in detail since more putative components of the JH signaling cascades, such as binding proteins and novel targets of JH intracellular receptors, putative JH membrane receptor, and targets of Kr-h1, need to be identified and characterized. Moreover, in *Drosophila*, Kr-h1 is mainly considered as the transcriptional repressor to antagonize 20E signaling, whereas it also functions as a transcriptional activator in the adult *Locusta migratoria* by recruiting CBP after phosphorylation (Wu Z. et al., 2021). The evolutionary conservation and more detailed analysis of transcriptional activation activity of Kr-h1 in *Drosophila*, and post-translational modification (Kim et al., 2021; Wu Z. et al., 2021), will further dissect the JH functions in directing insect development.

## Author Contributions

XZ and SNL drafted and wrote the manuscript and figures. SL provided conception, guidance, editing, and support with the manuscript. All authors contributed to the article and reviewed the final manuscript.

## Conflict of Interest

The authors declare that the research was conducted in the absence of any commercial or financial relationships that could be construed as a potential conflict of interest.

## Publisher’s Note

All claims expressed in this article are solely those of the authors and do not necessarily represent those of their affiliated organizations, or those of the publisher, the editors and the reviewers. Any product that may be evaluated in this article, or claim that may be made by its manufacturer, is not guaranteed or endorsed by the publisher.
